# Introduction of Varroa destructor has not altered honey bee queen mating success in the Hawaiian archipelago

**DOI:** 10.1038/s41598-020-80525-5

**Published:** 2021-01-14

**Authors:** Lauren M. Rusert, Jeffrey S. Pettis, David R. Tarpy

**Affiliations:** 1 Department of Entomology & Plant Pathology, North Carolina State University, Campus Box, Raleigh, 7613 USA; 2 Biology Graduate Program—Ecology & Evolution, North Carolina State University, Raleigh, NC USA; 3 Pettis and Associates LLC, Salisbury, MD 21801 USA; 4 Present Address: Department of Entomology, University of California Davis, Davis, USA

**Keywords:** Behavioural ecology, Molecular ecology

## Abstract

Beekeepers struggle to minimize the mortality of their colonies as a consequence of the parasitic mite *Varroa destructor* in order to maintain a sustainable managed pollinator population. However, little is known about how varroa mites might diminish local populations of honey bee males (drones) that might affect the mating success of queens. As one of the world’s last localities invaded by varroa mites, the Hawaiian Islands offer a unique opportunity to examine this question by comparing queens mated on mite-infested and mite-free islands. We raised queen bees on four Hawaiian Islands (Kaua‘i, O‘ahu, Maui, and Hawai‘i) and subsequently collected their offspring to determine queen mating frequency and insemination success. No significant difference for mating success was found between the islands with and without varroa mites, and relatively high levels of polyandry was detected overall. We also found a significant association between the number of sperm stored in the queens’ spermathecae and the number of managed colonies within the localities of the queens mated. Our findings suggest that varroa mites, as they currently occur in Hawai‘i, may not significantly reduce mating success of honey bee queens, which provides insight for both the reproductive biology of honey bees as well as the apiculture industry in Hawai‘i.

## Introduction

Island systems, such as the Hawaiian archipelago, offer a rare opportunity to study biological invasions and other dynamics of natural- and managed ecosystems. This chain of tropical islands is highly favorable to honey bees because the year-round abundance of nectar and pollen resources^1^ that facilitates a high density of feral colonies, and as a result there is a significant concentration of commercial queen producers in the state^2^. Hawai‘i is also one of the last locations on earth where honey bees were devoid of the devastating parasite *Varroa destructor* that has ravaged feral- and managed honey bee populations globally (with notable exceptions like Australia^3^). The parasite shifted hosts from *Apis cerana* to *A. mellifera* in the 1950s on two separate occasions in Russia and in Japan^4^. Unlike *A. cerana*, *A. mellifera* cannot as readily cope with the varroa mite, which feed on the fat bodies of developing pupae^5^, transmit viruses and pathogens^6^, and if left uncontrolled can weaken a colony to its collapse^7^. Varroa mite infestations can also be detrimental to honey bee physiology, including, but not limited to affecting their vitellogenin levels, hemocytes, ecdysteroid titers, and weight^8,9^. When varroa mites were first detected on O‘ahu in 2007 then on Hawai‘i Island (Big Island) in 2008^10^, the honey bee population in the archipelago was irrevocably changed. Despite the first detection of the mite to those islands, the other main islands—Maui, Kaua‘i, Moloka‘i, and Lāna‘i—remain free of varroa^10^, constituting an intriguing natural laboratory to study the effects of varroa parasitism on honey bees.

It is unclear if the increased colony losses of feral and managed honey bees as a consequence of varroa mites has led to a decrease in population density^6^. To date, there have been no documented recordings of such feral colony loss, and there has been only one such mention of managed hive decline since the arrival of the varroa mite to Hawai‘i (on the island of Oahu from 419 to 279 hives)^11^. In some rare instances, feral honey bee populations have persisted even with varroa mites present^12,13^. In other instances, honey bee feral populations have seen a dramatic decline because of mites^14,15^. Among other consequences, fewer colonies would presumably result in a decreased availability of honey bee males (drones), which could thereby significantly diminish the mating frequency of honey bee queens. In addition, the varroa mite’s preference for drone brood over worker brood^16,17^ may further hinder their population as well as colony health, leading to increased mite infestation levels and overall colony decline. It has been demonstrated that *Apis mellifera* queens typically mate with an average of ~ 12 drones^18,19^, and as such varroa mites may significantly impact the mating success of honey bee queens particularly in Hawai’i where they have been newly introduced.

Although mating frequency is one of the proxies that define queen bee reproductive potential (= quality), several other measures are frequently used to quantify their fecundity and putative fitness^20–22^. One suite of such measures is morphological analysis, including thorax width, head width, queen weight, and spermathecal diameter^23,24^. Morphological measurements are mainly an indication of a queen’s rearing environment—the age at which she is initially reared as a queen, the population and nutrition of the queen-rearing colony, and the incubation of her cell by worker bees—all of which can vary greatly and affect queen phenotype^25^. For example, Dedej et al.^26^ showed that queen larvae reared from first- and second instars had significantly increased ovariole numbers and other characteristics compared to those reared from later instars. A second suite of traits quantify insemination success, such as the number of spermatozoa stored within the spermathecae and their viability^27–29^. Insemination success is less of a factor of a queen’s colony environment but rather the local honey bee population. On average, a queen stores approximately 5–7 million sperm, and spermathecae with fewer than 3 million are considered to be poorly mated^30^. Sperm viability, on the other hand, is the percentage of stored sperm that is alive and can successfully fertilize an egg. Physical and mating quality are interdependent and positively correlated; it has been shown that queen body size is associated with mating success, with larger queens having higher sperm counts and mating with an increased number of drones^22,31^. Surveyed beekeepers consider a sperm viability of 80% or greater to be sufficient^29^. Although under-studied, other factors that affect sperm viability are temperature and pesticide exposure; when temperatures reach below 4 °C or above 40 °C for 1, 2, or 4 h, sperm viability decreases considerably^29,32^. In addition, it has been observed that different dosages of pesticides (e.g., the organophosphate coumaphos and the neonicotinoid imidacloprid) will decrease sperm viability in a spermatheca^33–35^. All of these queen reproductive quality measures help to assess the health of queen bees and aid in creating guidelines for overall colony sustainability.

Understanding the impact that mites have had on the reproductive quality of queen honey bees will provide crucial insight into underlying factors affecting honey bee colonies. In this study, queens were mated on four Hawaiian Islands, two with varroa (O‘ahu and Big Island) and two without (Maui and Kaua‘i), to quantify the negative impacts of *Varroa destructor* on queen reproductive quality.

## Materials and methods

### Experimental design

**Figure 1:**
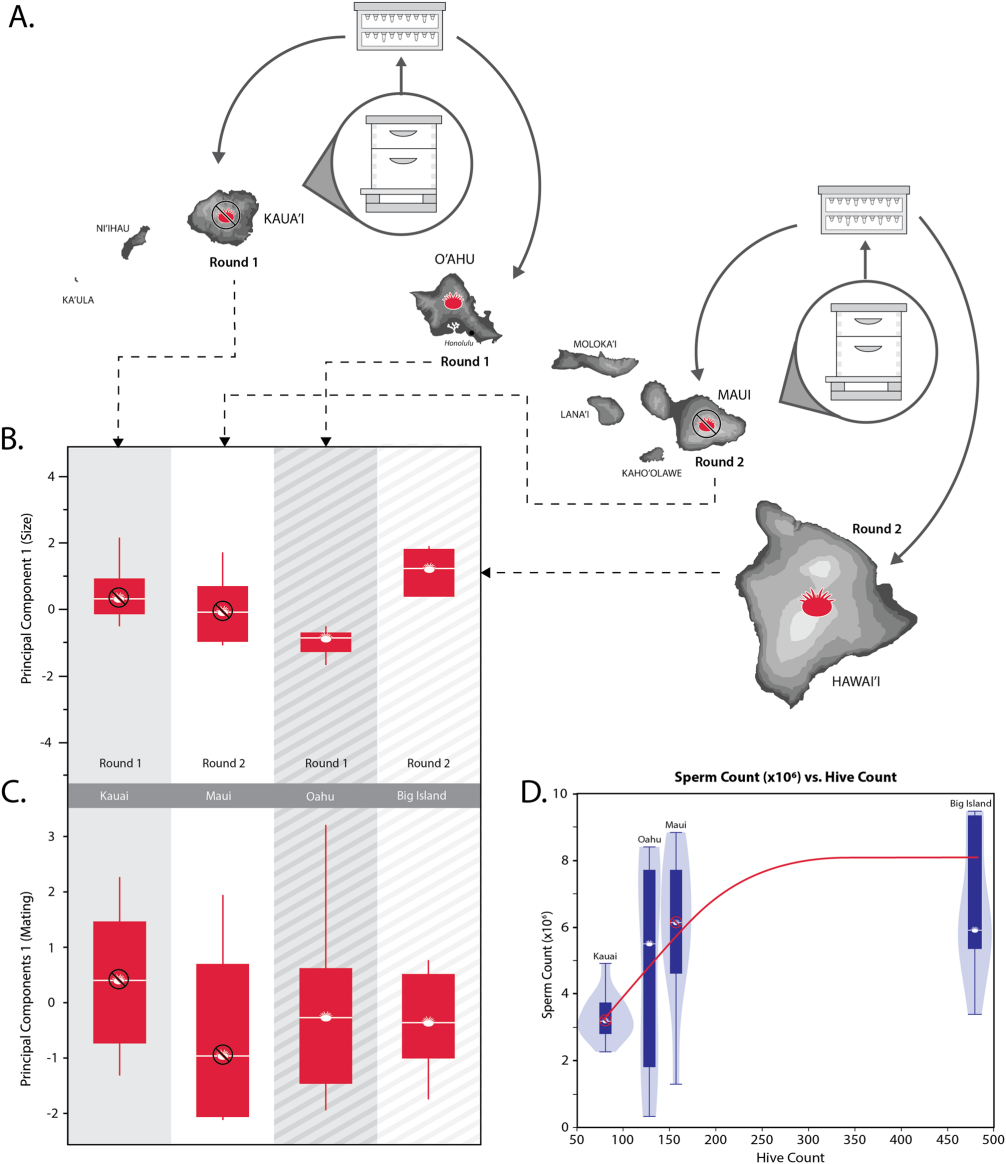
(**A**) Map of the Hawaiian Islands where the experimental queens were reared and mated. Two rounds of young worker larvae were separately raised as queens on varroa-free islands (Kaua‘i and Maui, respectively) then placed in nucleus hives on either the same island or a varroa-infested island (Hawai‘i and O‘ahu, respectively) for the queens to mate. (**B**) Principal Component 1 for queen body size—with loading variables of mean weight (mg), head width (mm), thoracic width (mm), and spermatheca diameter—were not significantly different among the queens mated on the four islands. (**C**) Principal Component 1 for mating quality—with loading variables of mean % sperm viability, sperm count (× 10^6^), observed mating number, and effective paternity frequency (*m*_*e*_)—were also not significantly different among the four populations of queens. (**D**) There was a significant correlation between queen stored sperm count (× 10^6^) and managed hive number within a 4.83 km (3-mile) radius of the apiaries where the queens were mated.

In April 2017, we established queen-breeding apiaries comprised of 20 nucleus hives on four Hawaiian islands—two with confirmed infestations of varroa mites (Hawai‘i or ‘Big Island’, and O‘ahu) and two without (Kaua‘i and Maui). Each nucleus hive consisted of 2–5 frames of bees (~ 3000–7500 adult workers), a laying queen, brood, pollen, and honey, which we established using local hive resources and bees. All nucleus colonies, including those on islands with varroa present, did not receive any synthetic miticides or chemical measures throughout the experiment. In May 2017, we grafted < 1-day old larvae from a single colony on Maui (in the town of Kihei) and placed them in an unrelated queenless colony where the bees were able to rear them as queens to adulthood^36^. Ten days after grafting, we removed the queen from 20 of the nucleus hives and introduced individual mature queen cells into them so that the newly emerged queens could emerge and mate. On the same day, we physically transported the remaining queen cells to Big Island as a personal item via a commercial flight (taking great care to keep them immobilized and warmly incubated), where we similarly introduced them into 20 queenless nucleus colonies in Hilo, HI (Fig. 1A). Two weeks later, we similarly grafted a second round of larvae from an unrelated colony on Kaua‘i (in the town of Lihue) and placed them into a separate queen-rearing colony. After 10 days, we introduced these cells into 20 queenless nuclei in the same apiary to allow them to emerge and mate. On the same day, we again personally transported the remaining queen cells to O‘ahu (in the town of Waimanalo) and placed them into queenless nuclei in that apiary (Fig. 1A). The transportation of queens ensured that larvae grafted from islands without varroa mites were introduced to islands with varroa as to not inadvertently introduce the mite, as well as to establish two biological replicates to control for maternal lineage. Once the queens had mated and started to lay eggs (roughly 4–6 weeks following the introduction of their cells), we collected ~ 200 emerging worker offspring from each surviving nucleus colony and stored them in separate vials of 70% ethanol. We used the worker samples for genotyping analysis to measure mating frequency of each queen. After the workers were collected, we shipped the worker samples and live queens to North Carolina State University for subsequent analyses, measuring the wet weight of each queen prior to shipping. Into each of the five queen “battery boxes,” we placed a single iButton data logger (Thermochron) with an operating range of − 40 to + 85 °C and accuracy of ± 1.0 °C recording at 5 min intervals during the entire transit time, simply to verify if there were any significant temperature fluctuations during shipping and statistically compensate for any abnormal shipping conditions.

### Morphological analyses

We photographed each queen under a dissecting microscope at 40 × for morphological measurements then decapitated her to euthanize. We separated the 5th and 6th abdominal sternites of each queen to reveal the spermatheca, which was removed, photographed, and immediately processed for sperm count and viability (methods below). We placed each queen’s head, thorax, and what was left of the abdomen into its own 0.5 mL microcentrifuge tube and frozen at − 80 °C for subsequent viral analyses (see below). We processed all images using JDraw calibrated to an image of a 1.00 mm glass ruler, measuring the head width, thorax width, and spermathecal diameter of each queen.

### Viral analysis

Because the queens were shipped live but the workers were preserved in ethanol, we only analyzed the queens for pathogens. Using established protocols^37–39^, we extracted RNA from each queen’s eviscerated body (head, thorax, and remaining abdomen) using a bead beater by first crushing each sample in individual microcentrifuge tubes, then adding 600 μL Trizol (Thermo Fisher Scientific, HQ in Waltham, MA, USA). We then extracted the RNA following the Trizol protocol (Thermo Fisher Scientific, HQ in Waltham, MA, USA). Once extracted, we tested the samples using the NanoDrop for quality and concentration. We normalized the RNA by diluting it with water to 200 ng/μL then synthesized the cDNA (Biobasic Inc. in Markham, ON, Canada) with BioBasic Reverse Transcriptase Mix in the thermocycler for 25 °C for 10 min, 42 °C for 15 min, 85 °C for 5 min, then held at 4 °C and diluted with 50 μL water. To detect the pathogens Nosema spp., trypanosome spp., acute bee paralysis virus (ABPV), black queen cell virus (BQCV), chronic bee paralysis virus (CBPV), deformed wing virus types A and B (DWV-A & DWV-B), Israeli acute paralysis virus (IAPV), and Lake Sinai virus (LSV), we performed reverse transcription quantitative PCR (RT-qPCR). Using SYBRGreen Mastermix (Thermo Fisher Scientific, HQ in Waltham, MA, USA), we performed qPCR, repeating it in triplicate on a 384-well QuantStudio Flex 6 plate (Thermo Fisher Scientific, HQ in Waltham, MA, USA) then analyzed the data using a dilution series to quantify the standard curve for the copy number. We used the Power-Up SYBRGreen protocol for the cycle settings, and we used the reference genes Actin, Apo28s, and GapDH to normalize the results through GeNorm.

### Semen analyses

Following protocols outlined in Lee et al.^37^, the spermathecae from each queen was placed into 1000 µl of saline buffer, ruptured with forceps to release sperm, gently mixed with forceps, and transferred via glass Pasteur pipet into an amber chromatography vial containing 10 µL of Syber 14 (Invitrogen Live/Dead sperm staining kit #L7011; 1 mM in DMSO) diluted 1:500 into Dimethylsulfoxide (99.8%) and 10 µL propidium iodide solution (2.4 mM in water). The vial was gently vortexed for 2 s at 2000 rpm to homogenize and mix. Vials were allowed to set a minimum of 5 min and a maximum of 4 h at room temperature to ensure uptake of the dyes into the cells. We processed each sample automatically using a Nexcelom Cellometer Vision cellular counter for which the exposure settings for fluorescence were 1200 ms for the Syber (live sperm) and 7000 ms for the propidium iodide (dead sperm). We counted each sample 3 × in different locations on the slide, and we averaged the resultant sperm counts and viability percentages to provide a single estimate for each queen.

### Genotyping analysis

We molecularly analyzed each worker by removing one hind leg, placing it in a 0.2 mL strip tube, and cutting it up with scissors that were ethanol and flame-sterilized between samples. DNA was extracted from each sample with either the Chelex 100 (BioRad) or HotSHOT protocols. For Chelex extractions, we ran a solution of 150 μL 5% Chelex 100 (BioRad) in dH2O, with 5 μL 10 mg/mL Proteinase K in a thermocycler for 60 min at 55 °C, 15 min at 99 °C, 1 min at 37 °C, 15 min at 99 °C, then held at 4 °C. For the HotSHOT extraction, we ran 75 μL alkaline lysis reagent (25 mM NaOH and 0.2 mM EDTA) in the thermocycler for 30 min at 95 °C, then held to cool at 4 °C. Once cooled, we added 75 µL neutralization buffer (40 mM Tris–HCL) to each sample. We subjected each sample to a single multiplex PCR to amplify seven microsatellite markers (A24, A76, A88, A113, Ap43, Ap81, and B124; see^28,40^). We combined 1.0 µL of the DNA extract with 5 µL multiplex PCR Kapa Master Mix, 0.67 µL tagged primers, and 3.33 µL DNASE/RNASE free H_2_O, totaling 10 µL. The solution was run in a thermocycler for 3 min at 95 °C, with 48 cycles of 15 s at 95 °C, 30 s at 57 °C, 45 s at 72 °C, with an elongation step of 5 min at 72 °C, then held at 4 °C.

For fragment analysis, we combined 1.0 µL of dilute PCR product with a 9.0 µL solution of Gene Scan Liz 500 sizing standard (Thermo Fisher) in Hi-Di Formamide (50 µL Liz 500 per 900 µL Formamide), denatured for 5 min at 95 °C, and sequenced on a 3730 DNA Analyzer (Applied Biosystems) by the NCSU Genomic Sciences Laboratory (Raleigh, NC). We analyzed mating number from microsatellite data using the program GeneMapper (Version 4, Applied Biosystems; www.thermofisher.com) then scoring the alleles visually. Once alleles were scored, the program COLONY^41^ analyzed the samples for the queens mating number (*N*_*o*_). Finally, we calculated effective paternity frequency^42^, the number of mates for a queen if all mates are represented equally.

### Statistical analyses

We analyzed each measure of queen body size using a fully factorial two-way ANOVA with Round (1 and 2) and Island type (with- or without varroa) as independent variables. Because all morphological measures were highly correlated, we employed a Principal Component Analysis (PCA) for size to generate the first principal component following Tarpy et al.^22^ that statistically combined queen weight (at 4–6 weeks post-commencement of egg laying, not after shipping and dissecting), head width, thoracic width, and spermathecal diameter. We similarly analyzed each measure of queen mating success using the same two-way ANOVA model, as well as conducted a second PCA on all of the measures of mating including sperm viability, sperm count, observed mating number (*N*_*o*_), and effective paternity frequency (*m*_*e*_). We subsequently employed regression to compare sperm count among the islands using local hive density as the independent variable (natural log transformed), which we obtained from the HI Department of beekeeper registry, 2017). All statistics were analyzed using JMP Pro v14.0 and reported with an α = 0.05, and all means are designated with ± SEM unless Agriculture (unpublished otherwise noted.

## Results

### Queen body size

For the 35 queens that were analyzed for queen reproductive quality, we found few significant differences for the various measures of body size. There were no significant differences among queens with respect to either thorax width (*F*_*3,34*_ = 1.13, *p* = 0.35) or spermatheca diameter (*F*_*3,34*_ = 1.08, *p* = 0.37). Although there were significant differences among queens for wet weight (*F*_*3,34*_ = 5.84, *p* < 0.005) and head width (*F*_*3,34*_ = 2.96, *p* < 0.05), these were driven by their interaction terms (both *F*_*1,34*_ > 3.34, *p* < 0.05). All measures were well within normal ranges of previous studies (wet weight: 199.9 ± 3.83 mg; head width: 3.7 ± 0.01 mm; thorax width: 4.8 ± 0.03 mm). However, when comparing overall body size using PC1 (Fig. 1B), we found no significant difference between Rounds (*F*_*1,34*_ = 1.04, *p* = 0.31) or between islands without (Kaua‘i and O‘ahu) and with (Maui and Big Island) varroa mites (*F*_*1,34*_ = 0.14, *p* = 0.71; power = 0.085, LSNumber = 458). This primary principal component, which was used as a combined proxy of overall body size, explained 60.3% of the total variation in the loading variables.

### Pathogen screen

We found surprisingly few pathogens among the queen samples. For all 38 queens that were analyzed across the four island populations, we detected none with the gut microsporidium Nosema (either *Nosema ceranae* or *N. apis*) or any trypanosome species. Similarly, no queen was detected to be infected with ABPV, BQCV, CBPV, IAPV, or any of the Lake Sinai viruses (LSV). Indeed, the only pathogens detected were the two forms of deformed wing virus (DWV-A and DWV-B)^11^. None of the queens that were reared and mated on Kaua‘i or Maui had positive detections of either DWV, and instead only queens mated on varroa-infested islands (O‘ahu and Big Island) were positive for DWV-A (n = 3) or both DWV-A and DWV-B (n = 5). We found that 50% of the eight queens mated on O‘ahu were virus free while the others were dual-infected (DWV-A: 322,954 ± 132,101 viral-genome equivalents per 200 ng/μL of RNA; DWV-B: 78,492 ± 74,075 genome equivalents per 200 ng/μL of RNA). For queens mated on Big Island, three had no DWV, three were infected with DWV-A only (185,665 ± 95,784 viral-genome equivalents per 200 ng/μL of RNA), and one was dual-infected (DWV-A = 1,947,843 and DWV-B = 92,934 genome equivalents per 200 ng/μL of RNA). Obviously, these results were statistically significant when comparing the incidence of both viruses between islands with and without varroa (DWV-A: Pearson χ^2^ = 15.5, df = 1, *p* < 0.0001; DWV-B: Pearson χ^2^ = 8.23, df = 1, *p* < 0.005) as well as their intensities (DWV-A: *t*_36_ = − 2.03, *p* < 0.05; DWV-B: *t*_36_ = − 2.49, *p* < 0.05).

### Insemination success

We found no significant differences among the queens for percent sperm viability (*F*_*3,34*_ = 0.99, *p* = 0.41). However, we did find a significant difference in stored sperm count from queens between the first and second rounds (*F*_*1,34*_ = 9.81, *p* < 0.005) but not between colonies with and without varroa (*F*_*1,34*_ = 2.11, *p* = 0.16). However, stored sperm count was most associated with the local managed beehive population (*F*_*1,36*_ = 10.4, *p* < 0.005; power = 0.881, LSNumber = 16.6). Figure 1D illustrates this positive association between sperm count (× 10^6^) and managed hive number (Ln scale) within a 4.83 km (3-mile) radius of the mating yards. Queens mated on Kaua‘i had the lowest mean sperm count (3.3 ± 0.61) with a hive number in the surrounding Lihue area of 80 hives, and those on O‘ahu with 5.0 ± 0.80 stored sperm and 127 hives in the Waimanalo area. The highest were those on both Maui (6.0 ± 0.67) with 156 hives in the Kihei area and queens on Big Island (6.0 ± 0.86) with 479 hives in the surrounding area.

### Mating number

We were only able to genotype a total of 28 colonies because several samples provided insufficient PCR amplification in order to score reliably. Of those analyzed, we scored an average of 47.5 ± 1.31 (SD) worker offspring per colony. We found no significant difference for observed mating number (*N*_*o*_) between the islands with varroa and islands without varroa (*F*_*1,24*_ = 0.39, *p* = 0.54). Similarly, we found no significant difference among the queens for effective paternity frequency (*F*_*3,24*_ = 1.20, *p* = 0.33) including those between varroa-infested and varroa-free islands (*F*_*1,24*_ = 0.38, *p* = 0.54). We detected relatively high polyandry overall, ranging from 25 to 38 subfamilies per colony. The mean *N*_*o*_ for queens on islands with varroa was 30.8 ± 1.40 and the mean for those on islands without varroa was 30.2 ± 1.08. By island, the average *N*_*o*_ for queens on Big Island (n = 6) was 31.3 ± 2.10, queens on Maui (n = 10) was 29.3 ± 0.63, queens on O‘ahu (n = 7) was 30.3 ± 1.94, and queens on Kaua‘i (n = 12) was 31.0 ± 7.00. When comparing overall mating success using the first principle component of a PCA, we found no significant difference between Rounds (*F*_*1,24*_ = 3.43, *p* = 0.08) or between islands with and without varroa mites (*F*_*1,24*_ = 0.00, *p* = 0.99; power = 0.054, LSNumber = 2878). PC1 for mating quality (Fig. 1C) explained 48.5% of the variation in the loading variables.

## Discussion

Our results confirm that there is high overall queen reproductive success on the four Hawaiian islands, irrespective of whether or not the varroa mite has been introduced to that sub-population. This demonstrates that even with the introduction of the most detrimental honey bee parasite—which contributes to many contributing factors to colony decline^43–47^—varroa mites do not seem to have a measurable impact on the mating success of queens and presumably the population of drones in this island locality. Our current estimates of mating number (mean ± SD: 30.9 ± 4.41) are on par with or even exceed the average mating frequencies for *Apis mellifera*^19^. Potential contributing factors for the high polyandry on all the Hawaiian islands may be a combination of factors, such as favorable weather, isolation from bee imports, the high abundance of feral and managed populations, higher tolerance of parasites, and year-round forage. It has been shown that even remote feral populations can have sufficient mating numbers^48^, so it seems that queens and drones are able to compensate even if local drone densities are relatively low.

Hawaii’s year-round favorable tropical weather may indicate that the regions where the queens mated were highly favorable to mating success^49^. The weather also lends itself to an increase in floral resources^1^, with Hawai‘i boasting as the highest-ranking annual honey production per hive at 59.4 kg (131 lbs) in 2017^50^. Higher resource abundance, particularly pollen, has been shown to be positively correlated with increased brood production^51^, leading to larger colonies. This increase in colony size often leads to an increase in queen-rearing and swarming, during which colonies experience a natural break in brood rearing thereby alleviating some effects of varroa-mite population growth^52,53^. Another outcome of swarming is an increase in the number of colonies, leading to a larger population of drones for which the queens to mate. An additional contributing factor as to why varroa may not be impacting honey bee mating in Hawaii’s environment compared to cooler climates may be because varroa mites feed on the fat bodies of bees^5^, thereby decreasing the ability for the bees to survive from their lipid stores over the winter. Indeed, according to the National Agriculture Statistics Survey in 2017, overwintering losses in Hawai‘i were at 1%, compared to the national U.S. average of 15%^50^.

While only a tangential focus of this study, our results from screening each queen for a suite of pathogens revealed some interesting trends. We verify that virus incidence and intensity are higher for individual bees when varroa mites are present in the local honey bee population^11^. Our findings cannot result from the vertical transmission of DWV (i.e., the mother queen from each round was infected and therefore inoculated her daughter queens through the egg^54,55^) because we would expect to see an equal number of DWV-infected queens mated on the varroa-free islands but instead we found none. It is possible but unlikely that we detected DWV in queens because their colonies were parasitized by varroa and indirectly infected the queens after they successfully mated. In the extreme circumstance, adult queens may also be parasitized by varroa^21^, but we cannot confirm that possibility in our study. While infected workers might have inoculated the queens through oral-oral trophallaxis, this would have had to occur within 4–6 weeks after the queens emerged. Since a queen’s pathogen profile does not mirror that of the workers after several weeks following her introduction to a new colony^39^, it seems less plausible that the queens were infected horizontally from worker feeding. Instead, our results strongly suggest that queens mated on varroa-infested islands were infected by mating with local DWV-infected drones. Sexual transmission of honey bee viruses has been demonstrated previously^55–57^, but our findings may suggest this route of exposure to be more common than previously recognized and may require additional investigation. While we cannot make strong inferences based on the limited sample size in this study, it does introduce a probing question about the pathogen ecology of queens and drones in the island archipelago and whether it is different from worker honey bees that have thus far been tested.

Following the first detection of the varroa mite to Big Island in 2008, over 100 feral hives were recorded within 2.59 km^2^ (one square-mile) in Hilo, HI. Although there are no records of this number being re-recorded or tested on any of the other islands, the possibility that there remains a high density of feral hives even with varroa mites introduced to Big Island and O‘ahu. In addition, the increase in mating frequency may be because of the strict regulations for honey bee importation to the Hawaiian Islands; bees and used bee equipment have been prohibited from entering the state for over 100 years^1^. The lack of constant introductions of new colonies may be preventing additional stressors being introduced into the honey bee populations of the Hawaiian Islands.

Perhaps not unsurprisingly, the physical measures of queen reproductive potential were not generally affected by different island environments; because of the genetic- and rearing sources were experimentally controlled, such results would be expected. To the contrary, sperm viability has been seen to decrease with extreme temperature exposure^29,32^. Due to the similar weather conditions among the islands, results for the viability are expected to be similar on each island. The only potential effects of shipping conditions in the current study would have been during transit to North Carolina, where we did not detect any prolonged temperature drops or spikes using the iButton thermal recorders (data not shown) or effects of deviant temperature fluctuations on sperm viability in the final results.

The most significant phenotypic difference in queen quality was in sperm count, with Big Island and Maui having the highest, followed by O‘ahu then Kaua‘i. We suspect this may be primarily a function of the number of managed hives surrounding the mating yards within a 4.83 km (3-mile) radius (HI Department of Agriculture; unpublished beekeeper registry, 2017). An increase in local population of managed hives may contribute to the overall increase in the nearby drone population, enhancing the likelihood that queens are fully inseminated. Although Big Island has the largest managed colony numbers in Hawai‘i (Fig. 1D), the number of feral colonies on Maui and Kaua‘i are slightly higher^58^, but these did not seem to have as much of an impact on the insemination success of queens on those islands.

Alternatively, the association between the number of managed beehives and insemination success may be an effect of drone size rather than population. Since Big Island has many large-scale queen producers^59^ who select for certain traits (particularly those from the “C” lineage; personal communication), drone size may be under passive selection as well. If these drones are larger, they may outcompete the smaller ones, leading to higher sperm counts in queens because larger drones have larger sperm counts^60^. To the contrary, many of the feral hives in the Hawaiian Islands are *Apis mellifera mellifera*^58^, a subspecies of honey bee that is slightly smaller and may therefore result in lower stored sperm counts in queens. Although mating frequency is high on all the islands, the sperm counts may give insight into a size differential of the drones on the different islands, since lower sperm counts and viability are associated with smaller and older drones, respectively^60,61^.

This study demonstrates that many factors may contribute to queen quality and mating success, but the negative effects of the varroa mite do not seem to impact the reproductive quality of queens in the sampled areas on the Hawaiian Islands. Nevertheless, because of limited accessibility, locations, and time, the sampled areas may not be fully representative for each island as a whole. More robust studies and monitoring need to be conducted on feral and managed honey bee colonies to quantify these dynamics over time.
